# Bilateral Pathways from the Basal Forebrain to Sensory Cortices May Contribute to Synchronous Sensory Processing

**DOI:** 10.3389/fnana.2018.00005

**Published:** 2018-01-23

**Authors:** Irene Chaves-Coira, Margarita L. Rodrigo-Angulo, Angel Nuñez

**Affiliations:** Department of Anatomy, Histology and Neuroscience, School of Medicine, Universidad Autonoma de Madrid, Madrid, Spain

**Keywords:** diagonal band of Broca, basal magnocellular nucleus, cholinergic neurons, somatosensory evoked potential, optogenetic stimulation, mouse

## Abstract

Sensory processing in the cortex should integrate inputs arriving from receptive fields located on both sides of the body. This role could be played by the corpus callosum through precise projections between both hemispheres. However, different studies suggest that cholinergic projections from the basal forebrain (BF) could also contribute to the synchronization and integration of cortical activities. Using tracer injections and optogenetic techniques in transgenic mice, we investigated whether the BF cells project bilaterally to sensory cortical areas, and have provided anatomical evidence to support a modulatory role for the cholinergic projections in sensory integration. Application of the retrograde tracer Fluor-Gold or Fast Blue in both hemispheres of the primary somatosensory (S1), auditory or visual cortical areas showed labeled neurons in the ipsi- and contralateral areas of the diagonal band of Broca and substantia innominata. The nucleus basalis magnocellularis only showed ipsilateral projections to the cortex. Optogenetic stimulation of the horizontal limb of the diagonal band of Broca facilitated whisker responses in the S1 cortex of both hemispheres through activation of muscarinic cholinergic receptors and this effect was diminished by atropine injection. In conclusion, our findings have revealed that specific areas of the BF project bilaterally to sensory cortices and may contribute to the coordination of neuronal activity on both hemispheres.

## Introduction

In the process of exploring their environment rats actively beat their whiskers although this process is not an isolated sensory stimulus. They analyze multiple contextual factors that may occur simultaneously in both whisker pads during their explorations. Thus, it would be expected that the primary somatosensory cortex (S1) in both hemispheres are activated and synchronized to analyze sensory inputs. It has been shown that the S1 neurons decrease their tactile responses when another somatosensory stimulus i.e., a distracter stimuli or sensory interference is applied simultaneously in the contralateral whisker pad, indicating that the S1 cortex receives information from the contralateral S1 cortex (Alenda and Nuñez, 2004). Therefore, the number of S1 neurons showing sensory interference decreases in animals with 192 IgG-saporin basal forebrain (BF) lesions that decreases the number of cortical cholinergic fibers. Thus, these data suggest that cholinergic projections may contribute to sensory interference (Alenda and Nuñez, 2007). In addition, it is well known that the cortex has the ability to focus sensory processing on selected sensory inputs while ignoring irrelevant inputs, involving the cholinergic system (Fanselow and Nicolelis, 1999; Reynolds and Desimone, 2003; Petkov et al., 2004; Sarter et al., 2005; Sussman and Steinschneider, 2006; Klinkenberg et al., 2010). These findings suggest that the cholinergic system may exert a modulation of both hemispheres in a coordinated way to enhance a relevant stimulus that may appear on either side.

The corpus callosum may also participate in the interaction between stimuli that occur on both sides of the body by precise projections between both hemispheres. Pyramidal neurons in layers 2/3 and layer 5 of S1 cortex target the contralateral S1 cortex via the corpus callosum projections and may therefore synchronize the activity of the two cortical hemispheres (Olavarria et al., 1984; Larsen et al., 2007; Petreanu et al., 2007; Aronoff et al., 2010). Furthermore, functional interactions between the S1 cortices of both hemispheres have also been suggested because a chronic suppression of the activity in one-hemisphere down-regulates the activity in the contralateral S1 cortex (Li et al., 2005). However, sensory interference was not affected after the corpus callosum transection (Alenda and Nuñez, 2007), which suggests that interhemispheric connections are not crucial for sensory interference.

Cholinergic projections to the cortex are provided by a dense innervation from disperse groups of cholinergic neurons within the BF. The BF contains cortically-projecting cholinergic and noncholinergic neurons as well as several interneurons (Zaborszky and Duque, 2000; Zaborszky et al., 2012). The BF includes the medial septum, the horizontal and vertical limbs of the diagonal band of Broca (HDB and VDB, respectively), the substantia innominata, and the nucleus basalis magnocellularis (B) nucleus (Semba and Fibiger, 1989; Semba, 2000; Zaborszky et al., 2012, 2015).

Anatomical studies have indicated the existence of a topographic organization of the BF efferent projections to the sensory cortices (Zaborszky, 2002; Zaborszky et al., 2005, 2015). Anatomical pathways linking the BF with sensory cortical areas studied in rodents have shown that separate or partially overlapping groups of BF neurons display specific projection pathways to primary sensory cortices of different modalities and to the prefrontal cortex (Semba, 2000; Zaborszky et al., 2015; Chaves-Coira et al., 2016). Optogenetic activation of cholinergic neurons in the BF facilitated somatosensory or auditory responses in S1 or in the primary auditory (A1) cortices (Chaves-Coira et al., 2016). Consistent with this specific organization of anatomical pathways, regionally-specific acetylcholine (ACh) release has also been demonstrated in visual and somatosensory cortices following the presentation of either visual or somatosensory stimuli, respectively (Fournier et al., 2004; Laplante et al., 2005). Therefore, distinct cholinergic BF neurons are theoretically capable of modulating specific cortical regions. Consequently, the BF cholinergic system possesses the necessary connectivity to modulate the cortex within the context of specific behavior thus contributing to the modulation of many brain functions.

However, the participation of BF cholinergic projections in coordinating the activity of two hemispheres has not been studied previously because it does not seem to have been supported by previous anatomical results. For example, it is reported that the projection from the B nucleus, which corresponds to the nucleus of Meynert in humans, to S1-M1 cortices is almost exclusively ipsilateral (Semba, 2000; Beak et al., 2010). However, these results contradict those of Katsumi et al. (1999) in which unilateral lesions of the B nucleus in rats decreased the cerebral metabolic rate of glucose in the ipsilateral frontal cortex, but recovered over the course of a few weeks. The authors suggested that this recovery could be due to the cholinergic projection from the contralateral nucleus because bilateral lesions of the B nucleus produced persistent bilateral suppression of glucose metabolism (Katsumi et al., 1999).

Since it is known that sensory processing is coordinated in both hemispheres, and that EEG changes that occur during the wake-sleep cycle are synchronized in both hemispheres, it is reasonable to believe that bilateral BF projections could contribute to these synchronous and integrated cortical activities. In the present study we examine whether certain populations of BF cells project bilaterally to sensory cortical areas, and provide anatomical evidence to support the important role of cholinergic projections in sensory integration.

## Materials and Methods

### Animals

Experiments were performed on 28 B6Cg-Tg (Chat-COP4*H134R/EYFP, Slc18a3)5Gfng/J mice (The Jackson Laboratory) of both sexes (3–6 months old). We used these transgenic mice because they express the light-activated cation channel, channelrhodopsin-2, tagged with a fluorescent protein (ChR2-YFP) under the control of the choline-acetyltransferase promoter (ChAT). Thus, all cholinergic neurons express the ChR2 and could be stimulated with blue light during optogenetic experiments. The animals were housed under standard colony conditions with food and water supplied *ad libitum*. All procedures were approved by the Ethics Committee of the Autonoma de Madrid University (CEI72-1286-A156), in accordance with Council Directive 2010/63 of the European Union. Efforts were made to minimize animal suffering as well as to reduce the number of animals used.

### Anatomical Procedures

**Figure 1:**
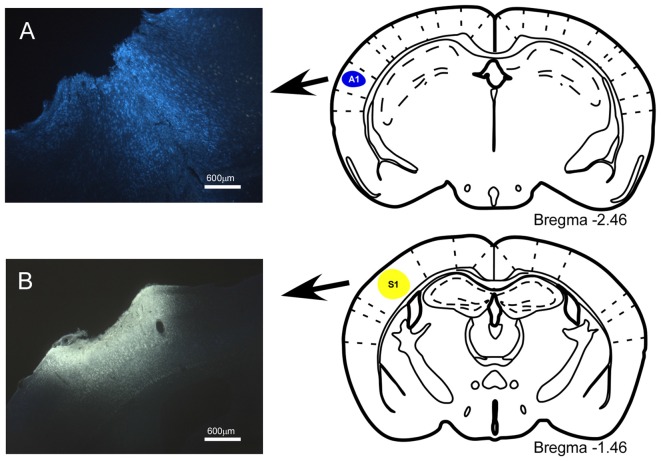
Location of injection sites into primary somatosensory (S1) and primary auditory (A1) cortices. **(A)** Schematic drawing and microphotograph of a brain coronal section showing the injection site in A1 cortex. **(B)** The injection site is located in S1 cortex.

The anatomical pathways linking the BF with cortical areas were studied by injecting, or depositing, the neuroanatomical fluorescent retrograde tracers Fluoro-Gold (FlGo; Fluorochromes, LLC., Denver, CO, USA) and Fast Blue (FB; Polysciences, Inc., Warrington, PA, USA). Solution of 4% FlGo was injected in the S1 using a 0.5 μl Hamilton syringe (20 nl applied slowly over a 2-min period; 10 nl per minute); alternatively, deposits of 2 mm^2^ absorbable gelatin “Spongostan” embedded in 1% saline solution of FB were placed in the A1 and the primary visual (V1) cortices (Figures 1A,B).

The animals were anesthetized with an intraperitoneal injection of ketamine (70 mg/Kg) plus xylazine (5 mg/Kg) before being placed in a stereotaxic frame for appropriate craniotomy; supplementary doses were applied when it was necessary (35 mg/Kg and 2.5 mg/Kg, respectively; i.p.). The analgesic Metacam (meloxicam1 mg/Kg; s.c) was also administered to the animals at the end of tracer injections. Animals were returned to the animal house under standard colony conditions.

### Injections/Deposits of Fluorescent Retrograde Tracers in Sensory Cortices

In all 18 animals, 20 nl of FlGo solution was injected through a Hamilton syringe in S1 at the following stereotaxic coordinates: antero-posterior, −1.7 mm from Bregma; lateral, 3.0 mm and vertical, 1.5 mm. FB deposits were placed for 20 min in 6 of the 18 animals in S1, at the same stereotaxic coordinates. In addition, FB deposits were placed in 6 of the 18 animals in A1 at the following stereotaxic coordinates: antero-posterior, −2.4 mm from Bregma; lateral, 4.0 mm and vertical, 2.2 mm; in the remaining six animals, FB deposits were placed in V1 at the following stereotaxic coordinates: antero-posterior, −6.3 mm from Bregma; lateral, 3.5 mm and vertical, 0.5 mm (Paxinos and Franklin, 2004).

Once the wounds had been sutured, the animals were housed in individual cages in accordance with the dimensions required for the species and located in a special post-surgery room at the Veterinary Office. The animals were treated with ibuprofen (Dalsy; 20 mg/cc solution; 3 cc/500 cc of drinking water) for the following days and additional doses of meloxicam, a non-steroidal anti-inflammatory drug (Metacam; 1 mg/Kg; s.c) was also administered when necessary.

After a survival period of 1 week the animals were anesthetized with an overdose of the same anesthesia and perfused transcardially with 4% paraformaldehyde in 0.1 M phosphate buffer at pH 7.3 followed by increasing concentrations of sucrose solutions (5%, 10%, 20%) in the same buffer. The brains were stored in 30% sucrose for at least 3 days for tissue cryopreservation to be frozen sectioned on the coronal plane at 40 μm. The sections were collected in three consecutively ordered series devoted to Nissl staining, fluorescent visualization and for ChAT immunostaining series. HDB and B nuclei were delimitated with the help of the adjacent Nissl stained sections and the use of the stereotaxic Atlas. In addition, in the sections devoted to fluorescent visualization the anterior commissure, third ventricle and caudate-putamen were taken as reference points for assessing the HDB correct location. Series processed for ChAT immunostaining sections were incubated with 1:100 goat anti-ChAT primary antibody and with 1:200 anti-goat Alexa 546 secondary antibody. The sections were mounted on glass slides, dehydrated through passage in ascending grades of alcohol, defatted in xylene for 30–60 min and finally coverslipped with DePeX mounting medium (Serva, Heidelberg, Germany).

Single and double-labeled neurons were studied under both a Nikon Axioskop fluorescent microscope and a confocal microscope (Spectral Leica TCS SP5) in which a Tile Scan tool of LAS AF software was used to acquire the images. Samples were analyzed using bio-mapping (maximal projections) by sequentially applying both lin405 mm (ultraviolet) laser line and linAr488 mm (applying argon) laser line, to ensure complete channel separation. The regions of interest were studied using 10×, 20×, 40× objectives and a 63× oil objective for the quantification of neurons in each channel. The images were a stack of sections in maximal projection, but the neurons were counted in each individual layer. Image stacks and maximal projections of the images were analyzed in the two channels (ultraviolet and green) and the merged image was also studied. The images shown in the figures are a stack of sections in maximal projection. For the semi quantitative study the neurons were counted in each individual layer of the confocal image.

### Optogenetic Stimulation and Electrophysiological Recordings

Experiments were performed on 10 adult (3–6 months-old) B6.Cg-Tg (Chat-COP4*H134R/EYFP, Slc18a3)5Gfng/J mice. The animals were anesthetized with an initial dose of ketamine (13 mg/kg) plus xylazine (5 mg/Kg) followed with isoflurane mixed with O_2_ (0.5%–1%; 0.5 l^−1^, min^−1^). The anesthetic level was monitored by toe pinch, respiration, and pupil dilation. Also, the anesthetic induced the presence of delta frequency waves (1–4 Hz) of high amplitude (>50 μV). The animals were placed on a water-heated pad (Gaymar T/Pump, Orchard Park, NY, USA) set at 37°C to maintain body temperature, and their head placed in a Kopf stereotaxic device (David Kopf Instruments, Tujunga, CA, USA) on which surgical procedures and recordings were performed. Skin incisions were infused with a local anesthetic (Lidocaine, 1%), and their eyes covered with mineral oil to prevent drying. After a midline skin incision, the periosteum and muscle were retracted to expose the skull. A craniotomy was then drilled and the duramater over the target areas was opened.

Field potential recordings were performed in S1 cortex (antero-posterior −1 mm to −2 mm, lateral 3 mm, vertical 1 mm from Bregma) through tungsten macroelectrodes (<1 MΩ, World Precision Instruments, WPI, Sarasota, FL, USA). Field potentials were filtered between 0.3 Hz and −100 Hz, and amplified using a DAM80 preamplifier (WPI). The signals were sampled at 1 kHz through an analog-to-digital converter built into the Power 1401 data acquisition unit, and fed into a PC computer for off-line analysis with Spike 2 software (Cambridge Electronic Design, Cambridge, UK).

Light stimulation of ChR2-expressing neurons was achieved with a light-emitting diode (LED; 473 nm; Thomas Recording, Germany) delivered from an optical fiber (core diameter 120 μm) positioned directly above the HDB or B nuclei. The LED was triggered with a single square-step voltage pulse (0.5 s duration) Illumination intensity was <30 mW/mm^2^, which is below the damage threshold of ~100 mW/mm^2^ for blue light (Cardin et al., 2010). Under these conditions, the effective stimulation area is assumed to be quite restricted as the total light energy transmitted within the cortical tissue *in vivo* decreases rapidly with distance (about 100–200 μm in radius; Aravanis et al., 2007).

### Sensory Stimulation

Whisker deflections were produced by brief air pressure pulses using a pneumatic pressure pump (Picospritzer; 1–2 kg/cm^2^, 20 ms duration), delivered through a 1-mm-inner diameter polyethylene tube. All whiskers were first trimmed to a length of 5 mm. The experimental protocol consisted of 120 air pulses delivered to the principal whisker at 0.5 Hz (4 min; control period) followed by the light stimulation. Air pulses at 0.5 Hz were delivered again to the selected whisker during 10 or 30 min after the optogenetic stimulation.

### Drugs

Atropine (1 mg/Kg in 0.9% NaCl i.p.) was administered 10 min before the start of recordings to assess whether the cholinergic modulation of the cortical responses was due to activation of the muscarinic receptors.

### Data Analysis

The average of the cortical evoked potentials in the S1 cortex triggered by tactile stimuli were calculated every 2 min (60 stimuli), using Spike 2 software. To perform statistical analysis, the area of the evoked potential was measured from the negative slope beginning with the first negative wave up to the same voltage level with a positive slope. The evoked potentials were recorded 4 min before blue light stimulation (the control period) and 10 or 30 min after the light stimulation. The magnitude of the change in the area was expressed as a proportion (%) of the base line control amplitude and plotted in function of time. The mean area of the control period (4 min) was considered 100%.

The results are reported as means ± SEM (Standard error of mean). Non-normally distributed data were compared with the Wilcoxon matched-pairs signed rank test. For multiple comparisons for normally distributed data (Shapiro-Wilk normality test), one-way analysis of variance (ANOVA) followed by Dunnett’s *post hoc* test was used. A *P*-value <0.05 was considered statistically significant. We have chosen this threshold to have a false positive risk probability less than 3:1.The data presented below had *P*-values less than 0.01, indicating that the probability of a false positive result is low. Graph Pad Prism 7 Software (San Diego, CA, USA) was used for the analysis.

## Results

### Bilateral Projections from BF Neurons to Sensory Cortices

**Figure 2:**
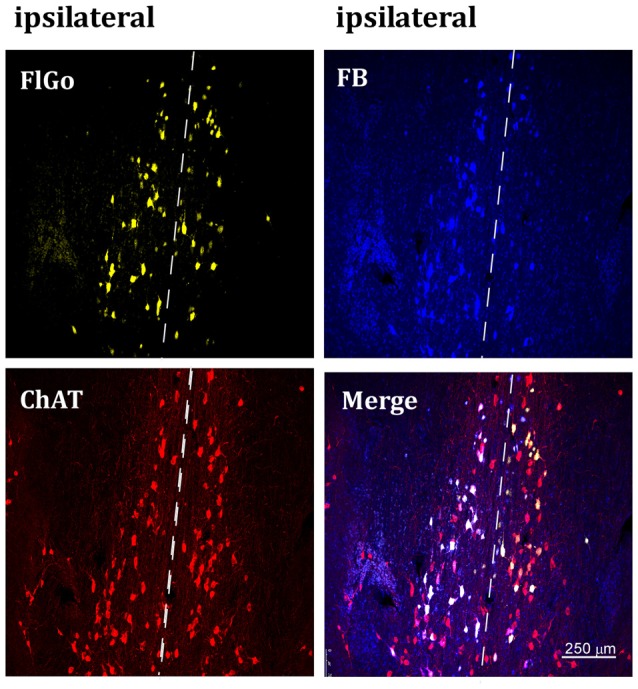
Confocal microphotograph of vertical limb of the diagonal band of Broca (HDB) showing labeled neurons of animals injected with the fluorescent tracers in the left hemisphere in S1 (yellow neurons) and A1 (blue neurons). Since the injection was in the left hemisphere, ipsilateral neurons are at the left side of the dash line (indicated above) and contralateral neurons are the ones located at the right side of the dash line indicating the middle line (upper images). Bottom left image shows positive neurons for choline-acetyltransferase (ChAT) immunostaining labeled in red color. FB: Fast Blue (blue) labeled neurons; Fluoro-Gold (FlGo; yellow): FlGo labeled neurons; ChAT (red): cholinergic labeled neurons. Labelled neurons were observed both in the ipsi (left hemisphere) and the contralateral (right) injection site.

**Figure 3:**
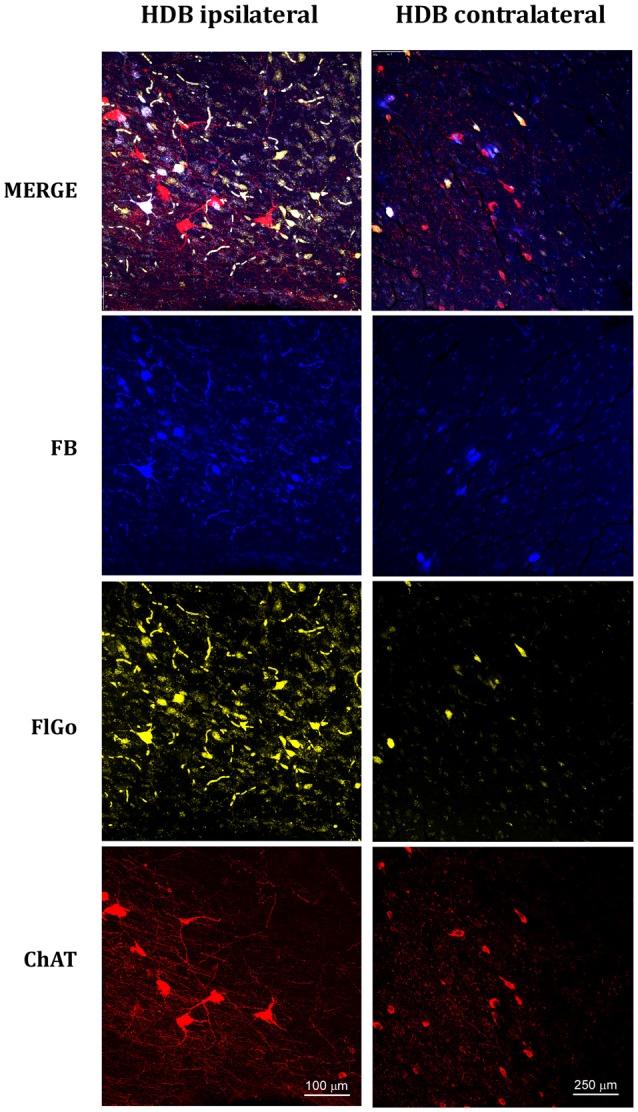
Confocal microphotographs of labeled neurons in horizontal limb of the diagonal band of Broca (HDB) nuclei of the basal forebrain (BF) of animal injected with both fluorescent tracers in the left hemisphere in S1 (yellow neurons) and A1 (blue neurons). The left column shows the HDB labeled neurons of the ipsilateral injection site; the right column shows the HDB contralateral labeled neurons to the injection site. Blue indicated FB labeled neurons; Yellow indicated FlGo labeled neurons and red color indicates positive neurons for ChAT immunostaining. The upper row shows the merge of all labeled neurons in HDB.

**Figure 4:**
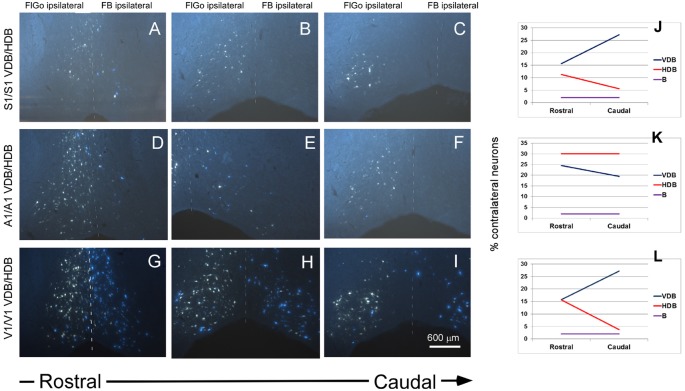
Fluorescent microscope images showing the rostro-caudal distribution of fluorescent labeled neurons. **(A–C)** Fluorescent microscope images from animals injected with FlGo and FB in S1. **(D–F)** Fluorescent microscope images from animals injected with FlGo in A1 and FB deposit in A1. **(G–I)** Fluorescent microscope images from animals injected with FlGo in V1 and FB deposit in V1. **(J–L)** Graphic representation of rostro-caudal distribution of neurons in VDB, HDB and B nucleus and the proportion of contralateral labeled neurons.

The anatomical pathways linking the BF with the cortical areas were studied by injecting, or depositing, neuroanatomical fluorescent retrograde tracers into the mice (Figure 1). The mice that received a fluorescent tracer injection in the S1 or A1 cortices showed labeled neurons in the ipsi- and contralateral BF. Stained neurons were present in the VDB (Figure 2), HDB (Figure 3) and SI areas but not in the B nucleus (<2% of labeled neurons). Similar projection patterns were observed in the VDB, HDB and SI when the retrograde tracer was applied to the A1 and V1 cortices (see Figures 4G–I). In all cases, the B nucleus showed an abundance of labeled neurons from ipsilateral trace injections, however, B neurons projecting to the contralateral sensory cortices were very scarce.

Although it is well known that BF cholinergic neurons project to sensory cortices (for review see Zaborszky, 2002; Woolf and Butcher, 2011), we corroborated these findings by performing an immunostaining ChAT study. Representative neurons in the VDB or HDB nuclei are shown in Figures 2, 3, respectively. The results indicate that most of the projecting neurons to the ipsi- and contralateral sensory cortices were cholinergic neurons.

In addition, a rostro-caudal gradient was observed in the distribution of contralateral BF projecting neurons, mainly in the VDB and HDB. S1 tracer injections in both hemispheres revealed ipsi- and contralateral projecting neurons at all levels of the VDB and HDB areas (Figures 4A–C). However, contralateral projection neurons showed a gradient in the rostro-caudal direction that increased from 16% to 27% in the VDB, while in the HDB the gradient decreased from 11% to 6% (Figure 4J). The gradient observed in the contralateral projections from the BF to the sensory cortices coincided with that observed in the ipsilateral projections (data not shown).

A1 tracer injections also showed ipsi- and contralateral labeled neurons in the VDB and HDB areas of the BF (Figures 4D–F). The proportion of contralateral labeled neurons was constant (30% of total neurons) at all levels of the rostro-caudal direction. However, a gradient was observed in the VDB (from 25% at rostral levels to 19% at caudal levels; Figure 4K). Ipsi- and contralateral labeled neurons were found in the VDB and HDB when retrograde tracers were injected in the V1 cortex (Figures 4G–I). Contralateral VDB labeled neurons (16%) were found at the rostral levels while 27% of labeled neurons were observed at the caudal levels when the tracer was located at the V1 cortex (Figure 4L). The HDB also showed a rostro-caudal distribution but in the opposite direction to the VDB, decreasing the percentage of contralateral labeled neurons from 16% to 4%. Similar to the S1, the gradient observed in the contralateral projections from the BF to the A1 or V1 cortices were similar to that observed in the ipsilateral projections (data not shown).

### Effect of Optogenetic Stimulation of HDB Neurons on Whisker S1 Cortical Responses

**Figure 5:**
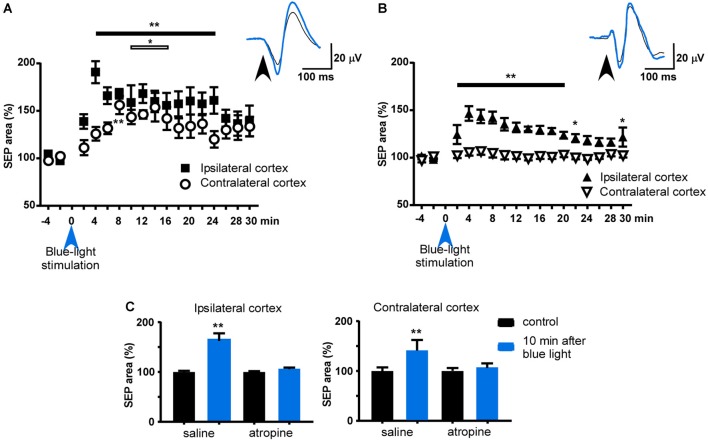
Effect of blue-light stimulation of HDB and B nuclei on ipsi- and contralateral S1 cortices. **(A)** Plot of the somatosensory evoked potential (SEP) area during the control period (4 min before blue-light stimulation) and 30 min after blue light stimulation of HDB. The mean area of the control period was considered as 100%. HDB induced a facilitation of both SEP recorded in both hemispheres, although the area increased slower in the contralateral cortex to the stimulated HDB. **(B)** Same plot as in **(A)** after blue light stimulation of B nucleus. Note that contralateral SEP area was not affected. **(C)** Plots of the effect of atropine sulfate on HDB stimulation. The effect was measured 10 min after HDB stimulation respect to the mean SEP area during the control period (4 min before blue light stimulation). After saline i.p. injection blue-light stimulation induced a facilitation of SEP area in both ipsi- and contralateral cortices (*n* = 8). However, the effect was blocked when atropine was i.p. injected 10 min before HDB stimulation. Insets in **(A,B)** show representative traces of the SEP in control and 10 min after optogenetic stimulation (black and blue tracers, respectively). *p < 0.05; **p < 0.01.

To test if the contralateral BF projections to the S1 cortex had the same functional effect on whisker responses as the ipsilateral projections, we applied a blue-light pulse to the HDB nucleus. Somatosensory evoked potentials (SEPs) were elicited with a short-lasting air pulse (20 ms duration) applied to a contralateral whisker of each of the S1 cortices. The effect of the blue-light pulse on whisker responses was simultaneously recorded in the S1 cortex of both hemispheres. The mean area of the earlier negative wave was calculated every 60 stimuli. The control period consisted of 4 min of continuous stimulation at 0.5 Hz and the mean area was considered to be 100%. Blue-light stimulation of the HDB induced an increase in the SEP areas in the S1 cortex of both hemispheres (Figure 5A). The SEP area increased rapidly when the blue-light stimulation was delivered to the HDB, and the SEP was recorded in the ipsilateral S1 cortex, reaching a maximum 4 min after blue-light stimulation (191 ± 12%, *P* = 0.0011; ANOVA plus Dunnett’s test; *n* = 8). The effect was sustained for up to 26 min after blue-light stimulation (142 ± 9%; *P* = 0.0033; ANOVA plus Dunnett’s test; *n* = 8). The SEP area slowly increased when the blue-light stimulation was delivered to the HDB, and the SEP was recorded in the contralateral S1 cortex, reaching a maximum 8 min later (156 ± 9%, *P* = 0.0017; ANOVA plus Dunnett’s test; *n* = 8) and sustained for up to 18 min after stimulation (142 ± 12%; *P* = 0.048; ANOVA plus Dunnett’s test; *n* = 8).

Blue-light stimulation of the B nucleus also induced an increase in the SEP area in the S1 cortex of the ipsilateral hemisphere, although to a lower extent or showing a less extended response, than when the light was delivered to the HDB (Figure 5B). The maximum effect was observed 4 min after blue-light stimulation (146 ± 8%, *P* = 0.001; ANOVA plus Dunnett’s test; *n* = 6). However, the SEP area was not affected when the blue-light stimulation was delivered to the B nucleus and the SEP recorded in the contralateral S1 cortex (*P* > 0.05; ANOVA plus Dunnett’s test; *n* = 6).

The facilitatory effect evoked by HDB stimulation was blocked by atropine sulfate (1 mg/kg; i.p.). In the control condition (after injection of saline solution; 0.1 ml) the SEP area of the ipsi- and contralateral S1 cortices increased to 168 ± 10% and 143 ± 7% (*P* = 0.0078, *P* = 0.0078, with respect to the control period; *n* = 8, in each group; Wilcoxon matched-pairs test; Figure 5C). Ten minutes after atropine application, blue-light stimulation did not affect the SEP area in either ipsi- or contralateral cortices 104 ± 2% and 106 ± 4% (*P* > 0.05, with respect to the control period; *n* = 8, in each group; Wilcoxon matched-pairs test; Figure 5C).

## Discussion

Our findings have revealed that the HDB and VDB nuclei of the BF project bilaterally to sensory cortices, facilitating whisker responses in the S1 cortex through activation of muscarinic receptors. By contrast, the B nucleus did not project bilaterally to the cortex. Previous data from our laboratory indicated that the HDB projects mainly to the S1 cortex, while the B nucleus does not show a specific pattern of cortical projections (Chaves-Coira et al., 2016). Taken together, these findings suggest that the areas of the BF that are mainly involved in the modulation of cortical sensory responses show bilateral projections to the cortex, while the B nucleus that has less effect on sensory modulation, only projects to the ipsilateral cortex. Consequently, it is possible that the bilateral projections from the BF may contribute to coordinating the neuronal activity of both hemispheres, and consequently enhance sensory processing.

The anatomical results presented here corroborated that the BF is a heterogeneous region that projects differently to the S1, A1 or V1 cortices according to the BF nuclei (VDB, HDB) and to the rostro-caudal distribution. This heterogeneity was also shown in the contralateral projections from the BF to the cortex. As is well known, the majority of the ipsi- and contralateral projecting neurons were cholinergic cells because they stained positively against the ChAT enzyme. There are numerous studies that have demonstrated the participation of cholinergic BF neurons in many behavioral functions such as learning, memory, attention and arousal (Buzsaki et al., 1988; Fibiger, 1991; Vanderwolf et al., 1993; Duque et al., 2000; Sarter and Bruno, 2000; Klinkenberg et al., 2010). For example, the detection of cues in attentional contexts depends on the cholinergic activity in the cortex (for review see Sarter et al., 2005). The release of ACh in the cortex increases prior to and during sustained attention tasks, with a further increase in response to distracters, presumably serving to enhance signals of behaviorally relevant targets (Himmelheber et al., 2000; Klinkenberg et al., 2010). Bilateral cholinergic projections from the BF may contribute to these behavioral functions.

Most of the previous anatomical studies have indicated that the BF projects to the ipsilateral cortex (see “Introduction” section). Using a combined method of retrograde tracing and optogenetic stimulation, we have shown that there is also an important contralateral projection that may contribute to the synchronization of the neurons located in the corresponding sensory areas of both hemispheres. The bilateral projections were restricted to the VDB and HDB nuclei but not to the B nucleus, indicating that the bilateral projections from the BF have a particular cortical projecting pattern for specific BF nuclei. The HDB nucleus shows specific anatomical projections, mainly to the S1 cortex that facilitate sensory responses (Chaves-Coira et al., 2016). By contrast, the B nucleus has more widespread targets in the sensory-motor cortex, and its effect on sensory responses is lower. Moreover, it has been reported that the projections from the B nucleus are almost exclusively ipsilateral (Semba, 2000; Beak et al., 2010; and present results). Consequently, optogenetic stimulation of the B nucleus only facilitated sensory responses in the ipsilateral cortex.

Taken together, these findings suggest that the BF nuclei that have specific projections to sensory areas, such as the VDB and HDB, have bilateral projections, while the less specific BF nucleus, such as the B nucleus, mainly project ipsilateral. Consequently, this suggests that bilateral projections may participate in sensory processing by maintaining a similar cortical activation level in the cortical areas of both hemispheres, helping to compare stimuli from both sides.

We used a transgenic mouse that expresses the ChR2 in cholinergic neurons to corroborate that most of the BF neurons projecting bilaterally to the cortex were cholinergic. Optogenetic stimulation of the HDB neurons evoked a long-lasting facilitation through the activation of muscarinic receptors since the effect was blocked by atropine. It has also been indicated that nicotinic cholinergic receptors might facilitate cortical responses (e.g., Howe et al., 2017). Although we cannot discard that they may participate in the initial phase of the facilitation response, our data suggest that the long-lasting facilitation was mainly due to activation of muscarinic receptors.

Neuronal synchronization is also an important tool for processing cortical information during these behavioral functions because it receives a vast amount of stimuli that have to be analyzed according to their relevance to a specific task or behavior. Task-relevant stimuli can synchronize activity between ensembles of cells (Engel et al., 2001; Fries et al., 2001; Buschman and Miller, 2007; Gregoriou et al., 2009). Likewise, fast-frequency oscillations have been observed during the performance of attentional task states (Fries et al., 2001; Bichot et al., 2005; Womelsdorf and Fries, 2006, 2007). Fast oscillations may synchronize activity in neural networks to support for example, cue detection. Recently, Howe et al. (2017) have shown that cues evoke phasic ACh release and an increased neuronal synchrony across several frequency bands in the prefrontal cortex. We propose that the bilateral projections described here may also contribute to the synchronization of rhythmic activities that may occur in both hemispheres.

In conclusion, our results together with previous findings, suggest that distinct cholinergic BF neurons are capable of participating in sensory modulation by means of a specific cortical projecting pattern. The BF cholinergic system has the connectivity needed to modulate the cortex within the context of the ongoing behavior, and contribute to sensory processing in a coordinated manner in both hemispheres.

## Author Contributions

MLR-A and AN conceived and supervised all aspects of the study. IC-C analyzed anatomical aspects of the data.

## Conflict of Interest Statement

The authors declare that the research was conducted in the absence of any commercial or financial relationships that could be construed as a potential conflict of interest.

## References

[B1] Alenda A. Nuñez A. (2004). Sensory-interference in rat primary somatosensory cortical neurons. Eur. J. Neurosci. 19, 766–770. 10.1111/j.1460-9568.2004.03150.x 14984427

[B2] Alenda A. Nuñez A. (2007). Cholinergic modulation of sensory interferente in rat primary somatosensory cortical neurons. Brain Res. 1133, 158–167. 10.1016/j.brainres.2006.11.092 17196557

[B3] Aravanis A. M. Wang L.-P. Zhang F. Meltzer L. A. Mogri M. Z. Schneider M. B.. (2007). An optical neural interface: *in vivo* control of rodent motor cortex with integrated fiberoptic and optogenetic technology. J. Neural Eng. 4, S143–S156. 10.1088/1741-2560/4/3/s02 17873414

[B4] Aronoff R. Matyas F. Mateo C. Ciron C. Schneider B. Petersen C. C. (2010). Long-range connectivity of mouse primary somatosensory barrel cortex. Eur. J. Neurosci. 31, 2221–2233. 10.1111/j.1460-9568.2010.07264.x 20550566

[B5] Beak S. K. Hong E. Y. Lee H. S. (2010). Collateral projection from the basal forebrain and mesopontine cholinergic neurons to whisker-related, sensory and motor regions of the rat. Brain Res. 1336, 30–45. 10.1016/j.brainres.2010.03.100 20381464

[B6] Bichot N. P. Rossi A. F. Desimone R. (2005). Parallel and serial neural mechanisms for visual search in macaque area V4. Science 308, 529–534. 10.1126/science.1109676 15845848

[B7] Buschman T. J. Miller E. K. (2007). Top-down versus bottom-up control of attention in the prefrontal and posterior parietal cortices. Science 315, 1860–1862. 10.1126/science.1138071 17395832

[B8] Buzsaki G. Bickford R. G. Ponomareff G. Thal L. J. Mandel R. Gage F. H. (1988). Nucleus basalis and thalamic control of neocortical activity in the freely moving rat. J. Neurosci. 8, 4007–4026. 3183710 10.1523/JNEUROSCI.08-11-04007.1988 PMC6569493

[B9] Cardin J. A. Carlén M. Meletis K. Knoblich U. Zhang F. Desselroth K. (2010). Targeted optogenetic stimulations and recording of neurons *in vivo* using cell-type-specific expression of Channelrhodopsin-2. Nat. Protoc. 5, 247–254. 10.1038/nprot.2009.228 20134425 PMC3655719

[B10] Chaves-Coira I. Barros-Zulaica N. Rodrigo-Angulo M. L. Nuñez A. (2016). Modulation of specific sensory cortical areas by segregated basal forebrain cholinergic neurons demonstrated by neuronal tracing and optogenetic stimulation in mice. Front. Neural Circuits 10:28. 10.3389/fncir.2016.00028 27147975 PMC4837153

[B11] Duque A. Balatoni B. Détári L. Zaborszky L. (2000). EEG correlation of the discharge properties of identified neurons in the basal forebrain. J. Neurophysiol. 84, 1627–1635. 10.1152/jn.2000.84.3.1627 10980032

[B12] Engel A. K. Fries P. Singer W. (2001). Dynamic predictions: oscillations and synchrony in top-down processing. Nat. Rev. Neurosci. 2, 704–716. 10.1038/35094565 11584308

[B13] Fanselow E. E. Nicolelis M. A. L. (1999). Behavioral modulation of tactile responses in the rat somatosensory system. J. Neurosci. 19, 7603–7616. 10460266 10.1523/JNEUROSCI.19-17-07603.1999 PMC6782523

[B14] Fibiger H. C. (1991). Cholinergic mechanisms in learning, memory and dementia: a review of recent evidence. Trends Neurosci. 14, 220–223. 10.1016/0166-2236(91)90117-d 1716012

[B15] Fournier G. N. Semba K. Rasmusson D. D. (2004). Modality and region-specific acetylcholine release in the rat neocortex. Neuroscience 126, 257–262. 10.1016/j.neuroscience.2004.04.002 15207343

[B16] Fries P. Reynolds J. H. Rorie A. E. Desimone R. (2001). Modulation of oscillatory neuronal synchronization by selective visual attention. Science 291, 1560–1563. 10.1126/science.1055465 11222864

[B17] Gregoriou G. G. Gotts S. J. Zhou H. Desimone R. (2009). High-frequency, long-range coupling between prefrontal and visual cortex during attention. Science 324, 1207–1210. 10.1126/science.1171402 19478185 PMC2849291

[B18] Himmelheber A. M. Sarter M. Bruno J. P. (2000). Increases in cortical acetylcholine release during sustained attention performance in rats. Brain Res. Cogn. 9, 313–325. 10.1016/s0926-6410(00)00012-4 10808142

[B19] Howe W. M. Gritton H. J. Lusk N. A. Roberts E. A. Hetrick V. L. Berke J. D.. (2017). Acetylcholine release in prefrontal cortex promotes gamma oscillations and theta-gamma coupling during cue detection. J. Neurosci. 37, 3215–3230. 10.1523/JNEUROSCI.2737-16.2017 28213446 PMC5373115

[B20] Katsumi Y. Hanakawa T. Fukuyama H. Hayashi T. Nagahama Y. Yamauchi H.. (1999). The effect of sequential lesioning in the basal forebrain on cerebral cortical glucose metabolism in rats. An animal positron emission tomography study. Brain Res. 873, 75–82. 10.1016/s0006-8993(99)01530-9 10433990

[B21] Klinkenberg I. Sambeth A. Blokland A. (2010). Acetylcholine and attention. Behav. Brain Res. 221, 430–442. 10.1016/j.bbr.2010.11.033 21108972

[B22] Laplante F. Morin Y. Quiron R. Vaucher E. (2005). Acetylcholine release is elicited in the visual cortex, but not in the prefrontal cortex by patterned visual stimulation: a dual *in vivo* microdialysis study with functional correlates in the rat brain. Neuroscience 132, 501–510. 10.1016/j.neuroscience.2004.11.059 15802200

[B23] Larsen D. D. Wickersham I. R. Callaway E. M. (2007). Retrograde tracing with recombinant rabies virus reveals correlations between projection targets and dendritic architecture in layer 5 mouse barrel cortex. Front. Neural Circuits 1:5. 10.3389/neuro.04.005.2007 18946547 PMC2526280

[B24] Li L. Rema V. Ebner F. F. (2005). Chronic suppression of activity in barrel field cortex downregulates sensory responses in contralateral barrel field cortex. J. Neurophysiol. 94, 3342–3356. 10.1152/jn.00357.2005 16014795

[B25] Olavarria J. Van Sluyters R. C. Killackey H. P. (1984). Evidence for the complementary organization of callosal and thalamic connections within rat somatosensory cortex. Brain Res. 23, 364–368. 10.1016/0006-8993(84)91270-8 6697197

[B26] Paxinos G. Franklin K. B. J. (2004). The Mouse Brain in Stereotaxic Coordinates. 2nd Edn. San Diego, CA: Academic Press.

[B27] Petkov C. I. Kang X. Alho K. Bertrand O. Yund E. W. Woods D. L. (2004). Attentional modulation of human auditory cortex. Nat. Neurosci. 7, 658–663. 10.1038/nn1256 15156150

[B28] Petreanu L. Huber D. Sobczyk A. Svodova K. (2007). Channelrhodopsin-2-assisted circuits mapping of long-range callosal projections. Nat. Neurosci. 10, 663–668. 10.1038/nn1891 17435752

[B29] Reynolds J. H. Desimone R. (2003). Interacting roles of attention and visual salience in V4. Neuron 37, 853–863. 10.1016/s0896-6273(03)00097-7 12628175

[B30] Sarter M. Bruno J. P. (2000). Cortical cholinergic inputs mediating arousal, attentional processing and dreaming: differential afferent regulation of the basal forebrain by telencephalic and brainstem afferents. Neuroscience 95, 933–952. 10.1016/s0306-4522(99)00487-x 10682701

[B31] Sarter M. Hasselmo M. E. Bruno J. P. Givens B. (2005). Unraveling the attentional functions of cortical cholinergic inputs: interactions between signal-driven and cognitive modulation of signal detection. Brain Res. Rev. 48, 98–111. 10.1016/j.brainresrev.2004.08.006 15708630

[B32] Semba K. (2000). Multiple output pathways of the basal forebrain: organization, Chemical heterogeneity, and roles in vigilance. Behav. Brain Res. 115, 117–141. 10.1016/s0166-4328(00)00254-0 11000416

[B33] Semba K. Fibiger H. C. (1989). Organization of central cholinergic systems. Prog. Brain Res. 79, 37–63. 10.1016/S0079-6123(08)62464-4 2685907

[B34] Sussman E. Steinschneider M. (2006). Neurophysiological evidence for context-dependent encoding of sensory input in human auditory cortex. Brain Res. 1075, 165–174. 10.1016/j.brainres.2005.12.074 16460703 PMC2846765

[B35] Vanderwolf C. H. Raithby A. Snider M. Cristi C. Tanner C. (1993). Effects of some cholinergic agonists on neocortical slow wave activity in rats with basal forebrain lesions. Brain Res. Bull. 31, 515–521. 10.1016/0361-9230(93)90118-u 8495376

[B36] Womelsdorf T. Fries P. (2006). Neuronal coherence during selective attentional processing and sensory-motor integration. J. Physiol. Paris 100, 182–193. 10.1016/j.jphysparis.2007.01.005 17317118

[B37] Womelsdorf T. Fries P. (2007). The role of neuronal synchronization in selective attention. Curr. Opin. Neurobiol. 17, 154–160. 10.1016/j.conb.2007.02.002 17306527

[B38] Woolf N. J. Butcher L. L. (2011). Cholinergic systems mediate action from movement to higher consciousness. Behav. Brain Res. 221, 488–498. 10.1016/j.bbr.2009.12.046 20060422

[B39] Zaborszky L. (2002). The modular organization of brain systems. Basal forebrain: the last frontier. Prog. Brain Res. 136, 359–372. 10.1016/s0079-6123(02)36030-8 12143394

[B41] Zaborszky L. Buhlm D. L. Pobalashinghamm S. Bjaaliem J. G. Nadasym Z. (2005). Three-dimensional chemoarchitecture of the basal forebrain: spatially specific association of cholinergic and calcium binding-protein-containing neurons. Neuroscience 136, 697–713. 10.1016/j.neuroscience.2005.05.019 16344145 PMC2080657

[B42] Zaborszky L. Csordas A. Mosca K. Kim J. Gielow M. R. Vadasz C.. (2015). Neurons in the basal forebrain project to the cortex in a complex topographic organization that reflects corticocortical connectivity patterns: an experimental study based on retrograde tracing and 3D reconstruction. Cereb. Cortex 25, 118–137. 10.1093/cercor/bht210 23964066 PMC4259277

[B40] Zaborszky L. Duque A. (2000). Local synaptic connections of basal forebrain neurons. Behav. Brain Res. 115, 143–158. 10.1016/s0166-4328(00)00255-2 11000417

[B43] Zaborszky L. Van den A. Gyengesi E. (2012). “The basal forebrain cholinergic projection system in mice,” in The Mouse Nervous System, eds Watson C. Paxinos G. Puelles L. (Amsterdam: Elsevier), 684–718.

